# Challenges that may impact achieving and maintaining accreditation in clinical laboratories in Zambia during the COVID-19 pandemic

**DOI:** 10.11604/pamj.2021.38.290.27836

**Published:** 2021-03-19

**Authors:** Victor Daka, Mutale Mubanga, Bright Mukanga, Ruth Lindizyani Mfune, Misheck Chileshe, Alfred Machiko, Steward Mudenda, Ephraim Chikwanda, Tobela Mudenda, Paul Simusika, Samson Mwale, Sinkala Musalula

**Affiliations:** 1 Public Health Department, School of Medicine, Copperbelt University, Ndola, Zambia,; 2 University of Zambia, School of Veterinary Medicine, Lusaka, Zambia,; 3 Centre for Infectious Disease Research in Zambia, Lusaka, Zambia,; 4 Mary Begg Health Services, Ndola, Zambia,; 5 University of Zambia, School of Health Sciences, Lusaka, Zambia,; 6 Tropical Diseases Research Centre, Ndola, Zambia,; 7 Ndola Teaching Hospital, Ndola, Zambia

**Keywords:** COVID-19, quality management system, accreditation

## To the editors of the Pan African Medical Journal

The coronavirus disease 2019 (COVID-19) which was first reported in Wuhan City, China, has spread to many countries worldwide. It was declared a global pandemic by the World Health Organisation (WHO) on 11^th^ March, 2020 [1]. As of 12^th^ January, 2021, a total of 89,707,115 cases and 1,940,352 deaths had been reported globally, while 28,596 cases and 471 deaths had been reported in Zambia [2].

A Quality Management System (QMS) can be defined as a coordinated set of activities to direct and control an organisation regarding quality. A laboratory QMS is cardinal for achieving desirable laboratory performance and assurance of good laboratory results [3]. Clinical laboratories in Zambia have been implementing laboratory QMS since 2010, and five (5) have attained ISO 15189 accreditation [4]. Implementing QMS in low resource settings such as Zambia presents with multiple challenges [5]. This has been compounded by the advent of the COVID-19 pandemic which has the potential to negate gains towards attaining and maintaining accreditation. Here we interrogate various challenges that may arise in achieving and maintaining laboratory accreditation amidst the COVID-19 pandemic and provide recommendations on possible mitigation measures.

Laboratory mentorship has been known to be a pivotal factor to the successful implementation of laboratory QMS. In Zambia, this model has mainly been supported by external cooperating partners [6]. However, due to COVID-19, travel restrictions and lockdowns have been implemented by many countries. This has the potential to restrict the availability of onsite QMS mentors.

Many countries depend on international trade for goods and services. The COVID-19 pandemic and the resulting global restrictions in air travel has negatively impacted the global supply chain [7]. Medical laboratories in Zambia depend on proficiency testing (PT) providers and laboratory supplies from companies that are not within the country to meet the requirements of ISO15189. Areas of QMS implementation that could be severely impacted are the importation of spare parts for equipment service, the supply of reagents, consumables, commercial control materials and calibrators.

The COVID-19 pandemic has greatly impacted health-care workers [8]. Staff in clinical laboratories have been reassigned to perform diagnostic duties related to the COVID-19 pandemic while others contracted the disease and were forced to go into isolation. The economic pressure experienced by the country and individual citizens including laboratory staff is likely to result in key laboratory staff changing their jobs. These (among other) lurking human resource problems are likely to exacerbate the pre-existing staff shortages, making it difficult for laboratories to adequately distribute staff assignments and meet the requirements of QMS implementation. Effects are likely to increase the turnaround time of tests, accelerate staff attrition leading to reduced laboratory productivity and an increase in the number of nonconformities. With the advent of the COVID-19 pandemic, many laboratories have had to include COVID-19 on their testing menu. COVID-19 has negatively impacted routine programs and the unique requirements of molecular testing protocols for COVID-19 has led laboratories to realign resources to meet the needs of increased testing [9].

The COVID-19 pandemic has resulted in a global recession with a severe impact on the economy of Zambia including funding challenges to the health sector [10]. Most laboratories in Zambia have relied on service engineers from other countries such as South Africa. Unfortunately, the availability of the service engineers and other requisite laboratory requirements amidst the COVID-19 pandemic may not be guaranteed.

### Recommendations

1) Increase local mentorship capabilities by training mentors drawn from accredited laboratories. Remote (offsite) mentorship using video conferencing tools such as Zoom, Microsoft Teams and similar applications should be explored. 2) Strengthen in-country training and certification of biomedical engineers to handle equipment maintenance and calibration needs of clinical laboratories. Traceability can be provided through training certification and externally calibrated reference equipment. 3) Laboratories should ensure the strict monitoring of reagent and consumable consumption data in order to have enough stock to sustain testing during unforeseen circumstances while procurement arrangements are being made. 4) In-country interlaboratory comparison should be encouraged through peer-to-peer assessments following the evaluation of possible referral laboratories. 5) Integrate COVID-19 testing in the QMS of laboratories to ensure quality. When changes in response to COVID-19 are planned and implemented, laboratories should ensure that the integrity of their QMS is maintained and that all applicable accreditation requirements are met. 6) Employment of medical laboratory personnel should be prioritised to meet the increased demand in testing. 7) There should be enough local funding for laboratory operations to cushion the impact of changing priorities by donors.

## Conclusion

The COVID-19 pandemic may affect QMS and ultimately healthcare in Zambia. There is need for targeted mitigation measures to ensure continual implementation of QMS and laboratory improvement.

## Authors’ conributions

VD and MM conceptualised the study, VD wrote the first draft. MM, BM, RLM, MC, AM, EC, TM, PS and SM reviewed and provided critical feedback on the manuscript. All authors read and approved the final manuscript.
